# Diffuse Large B-Cell Lymphoma: Clinical Presentation and Treatment Outcomes From the *OncoCollect* Lymphoma Registry

**DOI:** 10.3389/fonc.2021.796962

**Published:** 2022-02-02

**Authors:** Reena Nair, Dinesh Bhurani, Senthil Rajappa, Asha Kapadia, Rakesh Reddy Boya, Subramanian Sundaram, Hari Menon, Ganapathi S. Raman, Arun Seshachalam, Ramesh Nimmagadda

**Affiliations:** ^1^ Department of Clinical Hematology, Tata Medical Center, Kolkata, India; ^2^ Department of Haematology, Rajiv Gandhi Cancer Institute and Research Center, New Delhi, India; ^3^ Department of Medical Oncology, Basavatarakam Indo-American Cancer Hospital and Research Institute, Hyderabad, India; ^4^ Department of Medical Oncology, PD Hinduja National Hospital and Medical Research Center, Mumbai, India; ^5^ Department of Medical Oncology, Mahatma Gandhi Cancer Hospital and Research Institute, Visakhapatnam, India; ^6^ Department of Medical Oncology, V S Hospitals, Chennai, India; ^7^ Department of Medical Oncology, CyteCare Cancer Hospitals, Bengaluru, India; ^8^ Department of Medical Oncology, Kumaran Hospital (P) Ltd. (MCCF), Chennai, India; ^9^ Department of Medical Oncology, Dr GVN Cancer Institute, Trichy, India; ^10^ Department of Medical Oncology, Apollo Cancer Institute, Chennai, India

**Keywords:** lymphoma, diffuse large B cell, real-world evidence (RWE), anthracycline, rituximab, Middle Income Countries (MIC)

## Abstract

**Background:**

Diffuse large B-cell lymphoma (DLBCL) is the commonest subtype of lymphoma, standard CHOP was the treatment of choice, 42% of patients received rituximab, and 29% of patients were lost to follow-up during therapy, were reported in a study that collected retrospective data at 13 public and private hospitals for patients diagnosed with lymphoma between January 2005 and December 2009. The OncoCollect Registry was set up in 2017 to address the challenges in the collection of retrospective data through chart review, recording access to anthracycline and rituximab-based treatment, and to study outcomes and any improvement in the patient follow-up.

**Methodology:**

The OncoCollect Lymphoma group registry was set up at a national level with 9 participating centers. Lymphoma patients registered at these centers between 2011 and 2017 were included. The clinical features, prognostic stratification, associated comorbidities, response to first-line treatment, and 3-year outcomes of adult patients with DLBCL were analyzed.

**Results:**

Of the 5,886 lymphoma patients registered in the OncoCollect registry, 2,581 (44%) had DLBCL. A total of 1,961 were evaluable for frontline therapy. The median age at presentation was 57 years. Gender ratio was 1.6:1. At presentation, 43% were early stage, 70% had low and low intermediate IPI, 53% had extranodal disease, and 30.9% had one or more comorbidities (data available for 1,136 patients). The commonest extra nodal site was gastro-intestinal (23.98%) followed by head and neck (19.24%). The overall response rate was 79.29%. Complete remission was seen in 61.75%, partial response in 17.5%, stable disease in 4.3%, and progression in 7.9%. Patients who received anthracycline-based therapy (86.7%) and rituximab-based therapy (83.7%) had a 3-year event-free survival (EFS) of 69.67% and 68.48%, respectively. With a median follow-up of 33 months, the 3-year overall Survival (OS) and EFS were 75.37% and 66.58%, respectively.

**Conclusions:**

DLBCL remains the commonest (44%) lymphoma subtype and is curable with standard anthracycline- and rituximab-based therapies. The availability of rituximab has increased the proportion of patients receiving standard chemoimmunotherapy.

## Introduction

Diffuse large B-cell lymphoma (DLBCL) represents 30% to 40% of all cases of non-Hodgkin lymphoma (NHL) worldwide, with an estimated 150,000 new cases annually (1). Patients present with progressive lymphadenopathy, extranodal disease, or both and require therapy. More than 60% of patients are cured with chemoimmunotherapy. Cyclophosphamide, adriamycin, vincristine, and prednisolone along with rituximab (CHOP-R) remains the International standard for treatment-naive DLBCLs. CHOP along with rituximab (when feasible) is the standard first-line treatment in India (2–4). In patients with associated comorbidities and cardiac insufficiency, other alternative regimens include cyclophosphamide, etoposide, vincristine, and prednisolone along with rituximab (CEOP-R), CVP-R, or bendamustine and rituximab (BR), but there is no definitive “best regimen” defined. According to the LNH-98.5 trial, 15% of the patients present with primary refractory disease and 24% patients relapse during the 10-year median follow-up (5). Patients with treatment failure after CHOP-R have a poor outcome, in particular those with primary refractory disease.

Lymphoma patients are mostly treated in tertiary cancer centers and academic institutes. Some of these centers have electronic medical records (EMR) data of patient characteristics, diagnosis, admissions, medications, imaging, and laboratory data available for analysis. In the past, retrospective data collected mainly through chart reviews at 13 public and private hospitals suggested diffuse large B-cell lymphoma was the commonest subtype, standard CHOP was the treatment of choice, 42% received additional immunotherapy with rituximab, and 29% patients were lost to follow-up during treatment or postcompletion of therapy (6).

The OncoCollect Lymphoma Registry was set up in 2017 as a collaborative group effort to evaluate current practices in the management of DLBCLs in a middle-income country setting, to identify patterns of treatment and challenges in the rituximab era.

## Objective

The primary objective was to study the clinical presentation and outcomes of first-line therapy in DLBCL patients. The secondary objectives were to classify DLBCLs based on the WHO classification, understand the current patterns of treatment, and look at the feasibility of collaborative data collection.

## Methodology

**Figure 1:**
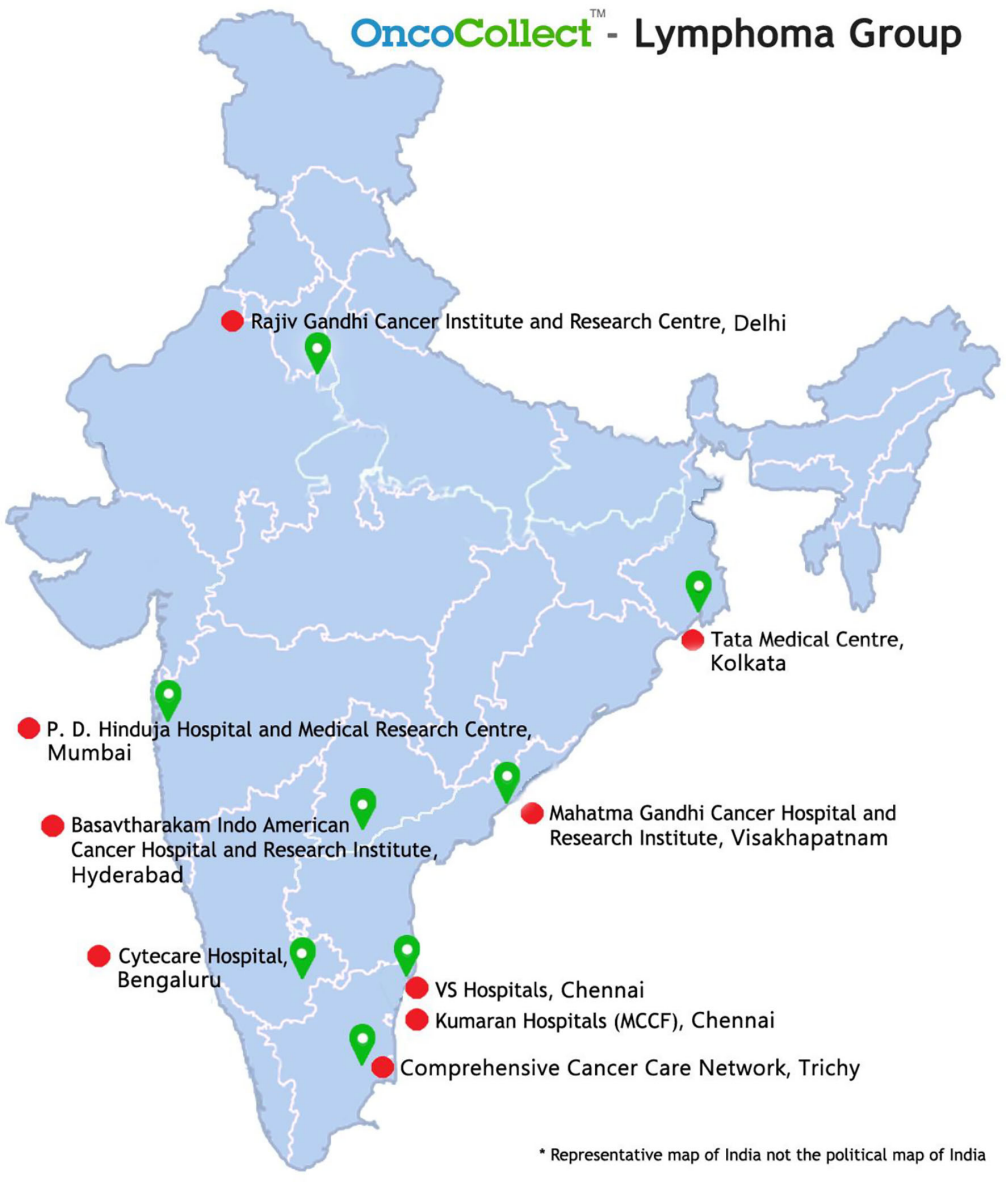
The distribution of institutes participating in the OncoCollect Lymphoma group.

This study was designed as a retrospective, multi-institutional, observational study. The registry was set up in 2017 and has 9 participating centers across the country till date (**Figure 1**). Both academic and community practices have contributed patient data to this Registry. OncoCollect software developed by Ramesh Nimmagadda Cancer Foundation (RNCF) was used to collate data. This study was approved by the Hospital Ethics Committee (HEC) of all participating institutes. A consent waiver was granted by the HEC.

A total of 5,886 patients (≥18 years) with a diagnosis of lymphoma were registered in the OncoCollect registry from 2011 to 2017. 1,961 treatment-naive DLBCL patients were considered evaluable for first-line treatment. One hundred eight primary central nervous system lymphoma (PCNSL) and 512 patients presenting after frontline therapy or with less than 4 visits in the outpatient clinic with no definite treatment prescribed at the participating center were considered second opinion seekers and have not been included for analysis.

### Disease Assessment

Patient details at diagnosis including clinical presentation, medical history, comorbidities, laboratory tests, treatment, and related toxicity were obtained from the hospital EMR. The histopathological diagnosis was reviewed at the participating center for most patients (subject to slide and block availability) prior to start of therapy. All DLBCL subtypes described in WHO (7) were included for analysis with the exception of PCNSL. DLBCL subtyping into germinal center B cell (GCB) or non-GCB was based on immune histochemistry (IHC) using Han’s algorithm (8). In patients with incomplete information, CD10-positive DLBCLs were classified as GCB and MUM-1-positive DLBCLs as non-GCG. Proliferative index (Ki-67), bcl-2, and IHC for C-myc were done to differentiate DLBCL from aggressive lymphomas. Fluorescence *in situ* hybridization (FISH) for C-myc was used to confirm Burkit and Burkit-like lymphoma.

Clinical variables recorded from the EMR included age, gender, Eastern cooperative oncology group (ECOG) performance status (PS), fever (>38.6°C), weight loss (>10% of body weight in 6 months), Ann Arbor stage, and presence of bulky disease (≥10 cm). Preexisting comorbidities were also recorded. As part of staging evaluation, PET-CT or plain CT imaging of the thorax and abdomen along with bone marrow biopsy was done. CSF cytology at diagnosis was done for patients with high risk of central nervous system involvement (CNS) or with symptoms and signs of CNS involvement. Laboratory test results included absolute blood counts, creatinine, albumin, and LDH. Hypoalbuminemia was defined as albumin level <3.5 g/dl. The cutoff for hemoglobin was 10 g/dl. The International prognostic index (IPI) based on the following criteria, i.e., PS (0–1 or >1), Ann Arbor stage (localized vs. extensive), extranodal site (0, 1 vs. >1), and LDH (normal vs. upper normal value) was calculated for patients (9).

### Treatment, Response, and Safety

The choice of therapy depended on patient’s general condition, comorbidities, available financial and social support, and institute preference. Prephase chemotherapy with steroids alone or with cyclophosphamide and vincristine (CVP) was given to patients with PS >2 at presentation, high LDH and advanced stage disease at risk of developing tumor lysis syndrome. Patients were grouped into 3 depending on the treatment they received, and these decisions were at physician’s discretion. Group 1 received standard chemoimmunotherapy CHOP+R-like regimen or infusional daEPOCH-R (for patients with high IPI, high c-myc expression on IHC, or proven double-hit lymphoma). Group 2 received CHOP-like chemotherapy without rituximab. Group 3 included all other regimens such as low-dose oral chemotherapy (steroids, cyclophosphamide, and etoposide), along with supportive care for frail elderly patients and advanced disease with organ failure, and nonanthracycline combination chemotherapy (CVP/CEOP+/−R, B-R), when physicians considered cardiotoxicity a limiting factor.

Early-stage (I and II) patients received ≤4 or 6 cycles of chemotherapy followed by consolidation radiotherapy as per institute policy. Advanced stage disease was treated with 6 or more cycles of therapy followed by radiotherapy to the site of initial bulky tumor or for partial response at the discretion of the local radiation oncology specialist.

The efficacy of treatment was assessed according to the National Cancer Institute-sponsored International Working Group criteria (10).

Response assessments were done mid-cycle and at the end of treatment with CT scans or PET-CT scan. Patients who died prior to mid-cycle evaluation, stopped treatment due to grade 4 morbidity or were lost to follow-up have been considered nonevaluable for response assessment. For patients who progressed on treatment or stopped follow-up for any reason post-mid-cycle assessment, the mid-treatment response is reported. The end-of-treatment response was reported for all other patients who completed treatment. End-of-treatment response evaluation by means of PET-CT scan was interpreted according to Deauville 5-point scale, with uptake in the mediastinum and liver used as a reference point. A score of 1 to 3 is considered to indicate a complete metabolic response (11).

Treatment-related toxic effects reported in EMR leading to hospitalization were analyzed. Reasons for death have been classified in three groups as follows: progressive disease, treatment toxicity, and other causes.

Follow-up of patients was obtained from the EMR records or by keeping a close contact (telephone/mobile) with the patient/family. Detailed physical examination, blood counts, and LDH were repeated on follow-up visits in most centers. Imaging studies for surveillance were as per the institute policy. Patients with residual disease and following relapse after first-line therapy were offered salvage chemotherapy and high-dose chemotherapy as consolidation (when financially feasible). Patients in remission were censored at the last follow-up. Patients who discontinued therapy at relapse and were on best supportive care at the time of last follow-up were considered deceased for the purpose of survival analysis.

### Statistical Analysis

Descriptive analysis was undertaken. Continuous variables are summarized as median, interquartile distance, or mean and standard deviation (SD). Categorical variables are expressed as absolute and percentage frequencies. Categorical covariates are compared using Chi^2^ test. The survival functions have been calculated and plotted using the Kaplan-Meier method, and the survival rate at 3 years of follow-up is reported with the estimated 95% confidence interval (95% CI).

The prognostic effect of covariate has been estimated using the Cox proportional hazard (PH) regression model, and reported as hazard ratio (HR) with 95% CI. A multivariate analysis for age, stage, IPI, treatment group, and use of anthracycline as well as rituximab was conducted.

All statistical analysis was done using R Open statistical software linked to the OncoCollect software.

## Results

The clinical characteristics of 1,961 DLBCL patients are summarized in **Table 1**. The median age was 57 years for the cohort. The gender ratio was 1.6:1. Initial clinical presentation includes the following: early stage (1 and 2) in 842/1,961 (43%), 87/842 (10.33%) early-stage patients had bulky disease, low and low-intermediate IPI in 1,328/1,895 (70%), and extranodal disease in 1,055/1,961 (53%). The common extranodal sites were gastrointestinal in 253 (23.98%), head and neck in 203 (19.24%), lung in 81 (7.67%), and genitourinary in 66 (6.25%). Bone and marrow uptake was present in 228 (21.61%). The cell of origin (COO) on block review was available for 950 patients, 438 GCB (47%), and 512 non-GCB (53%).

**Table 1:** Clinical characteristics at presentation, treatment, and response of 1,961 DLBCL patients.

	Number	Percent
**Age** (median (range)]	**57 years (18-89)**	
<65	1,403	71.55%
≥65	558	28.45%
**Gender** (ratio)	1.6:1	
Male	1,210	61.70%
Female	751	38.30%
**Stage**		
1	283	14.43%
2	559	28.51%
3	501	25.55%
4	618	31.51%
**IPI score** ^a^ #1,895		
Low	829	43.75%
Low-intermediate	499	26.33%
High-intermediate	344	18.15%
High	223	11.77%
**B’ symptoms** ^a^ #1,959		
Present	847	43.24%
**Extranodal site**		
Present	1,055	53%
**Sites**		
Gastrointestinal	253	23.98%
Head and neck	203	19.24%
Genitourinary	66	6.25%
Thyroid	44	4.17%
Lung	81	7.6%
Bone	228	21.61%
Others	180	17.06%
**Comorbidities** ^a^ #1,136		
No	785	69.10%
Yes	351	30.90%
**DLBCL subtype** ^a^ #950		
GCB	438	46.1%
Non-GCB	512	53.89%
**Chemotherapy regimen**		
Group 1	1,439	73.38%
Group 2	263	13.41%
Group 3	259	13.21%
**Anthracycline-based regimen**		
Yes	1,702	86.79%
No	259	13.21%
**Rituximab-based therapy**		
Yes	1,642	83.73%
No	319	16.27%
**1st-line best response**		
Complete response	1,211	61.75%
Partial response	344	17.54%
Stable disease	85	4.33%
Progression on treatment	166	8.47%
Nonevaluable	155	9.90%

a Missing value.

Bold values indicate subgroups.

Documentation of comorbidities was available in 1,136 EM records. One or more comorbidities were present in 351 (30.8%). Diabetes mellitus in 211 (18.57%) followed by hypertension in 158 (13.9%), and hypothyroidism in 33 (2.90%) were the commonest associated comorbid diseases. Blood-borne virus serology revealed HBsAg in 9 (0.79%), HCV in 2 (0.18%), and HIV seropositivity in 15 (1.32%).

**Figure 2:**
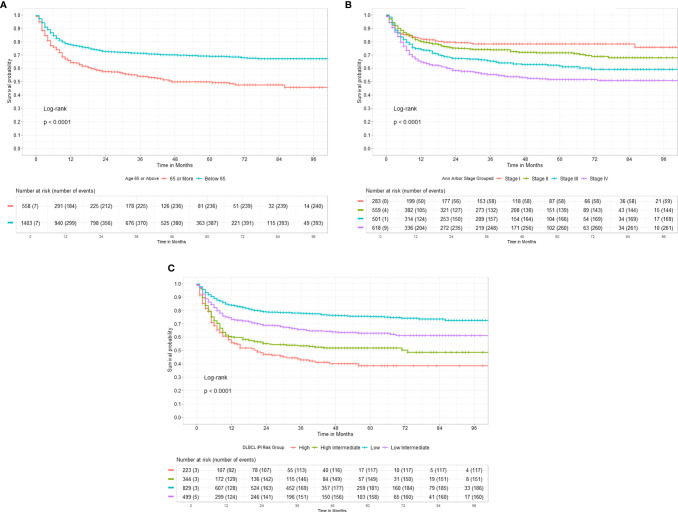
**(A)** Three-year Event Free Survival according to Age of DLBCL patients at presentation. **(B)** Three-year Event Free Survival according to Stage of DLBCL at presentation. **(C)** Three-year Event Free Survival according to International Prognostic Index risk group at presentation.

Data for age, stage, and IPI (**Figures 2A–C**) had a significant impact on the 3-year EFS while comorbidities at presentation and GCB subtype **(**mainly based on CD10 positivity) had no impact on 3-year EFS, as shown in **Table 2**.

**Table 2:** Three-year event-free survival outcomes of DLBCLs.

	3-Year EFS	95% Confidence Incidence	*p*-value
**Age group**			
<65 years	71.31%	68.85%–73.85%	
≥65 years	53.96%	49.56%–58.74%	<0.0001
**Stage**			
I	78.22%	73.37%–83.39%	
II	73.89%	70.11%–77.87%	
III	65.39%	61.07%–70.02%	
IV	55.40%	51.33%–59.80%	<0.0001
**International Prognostic Index (IPI)**			
Low	77.85%	74.94%–80.89%	
Low-intermediate	65.75%	61.36%–70.46%	
High-intermediate	53.52%	48.16%–59.48%	
High	43.00%	36.38%–50.81%	<0.0001
**Cell of Origin**			
Germinal center B cell (GCB)	68.32%	63.87%–73.07%	
Non-GCB	66.50%	62.30%–70.97%	0.53
**Comorbidities**			
No	67.42%	63.99%–71.05%	
Yes	66.91%	61.87%–72.37%	0.65
**Treatment**			
Group 1	71.33%	68.91%–73.83%	
Group 2	60.25%	53.97%–67.25%	
Group 3	43.81%	37.35%–51.39%	<0.0001
**Anthracycline-based therapy**			
No	43.81%	37.35%–51.39%	
Yes	69.67%	67.4%–72.03%	<0.0001
**Rituximab**			
No	56.22%	50.36%–62.76%	
Yes	68.48%	66.14%–70.91%	<0.0001
**Early-stage treatment**			
≤4 cycles	33.94%	25.26%–45.61%	
≤4 cycles + radiotherapy	60.55%	47.81%–76.67%	
6 cycles	85.30%	82.01%–88.72%	
6 cycles + radiotherapy	75.99%	68.45%–84.36%	<0.0001
**3-year EFS**	**66.58%**	**64.38%–68.84%**	
**3-year overall survival**	**75.37%**	**73.25%–77.55%**	

Bold values indicate subgroups.

### Treatment Efficacy

Patients were grouped depending on the treatment they received. Group 1 #1,439 patients received both anthracycline and rituximab (CHOP-R #1,334 and daEPOCH-R #105). Group 2 #263 patients received CHOP-like therapy without rituximab. Group 3 #259 patients received nonanthracycline-based treatment (CVP+/−R #96, CEOP+/−R #87, B−R #42, oral palliative chemotherapy #21, single-agent rituximab #7, and others #6). The distribution of treatments according to the predefined subgroups is summarized in **Table 1**. Patients received a median of 6 cycles (range 1–8) of chemotherapy. Rituximab was added to chemotherapy in 1,642 (83.73%) of the patients.

After first-line treatment, complete response (CR) was achieved in 1,211 patients (61.75%), partial response (PR) in 344 (17.54%), and stable disease in 85 (4.33%) after the first-line chemotherapy. Progression on treatment was seen in 166 (8.47%). A total of 155 patients (7.9%) could not be evaluated for response due to early mortality in 31 (1.5%), severe morbidity resulting in treatment dropouts, or failure to take treatment for financial reasons in 124 (6%). The CR rate in the 3 treatment groups was 975/1,439 (67.76%), 130/263 (49.43%), 106/259 (40.93%) for groups 1, 2, and 3, respectively. In total, 1,322/1,403 (94.23%) patients below 65 years received CHOP-like therapy, while 178/558 (31.90%) patients ≥65 years received group 3 regimens.

The 3-year EFS for the treatment groups 1, 2, and 3 was 71.33% [95% CI, 68.91%–73.83%], 60.25% [95% CI, 53.97%–67.25%], and 43.81% [95% CI, 37.35%–51.39%], respectively, as shown in **Table 2**.

**Figure 3:**
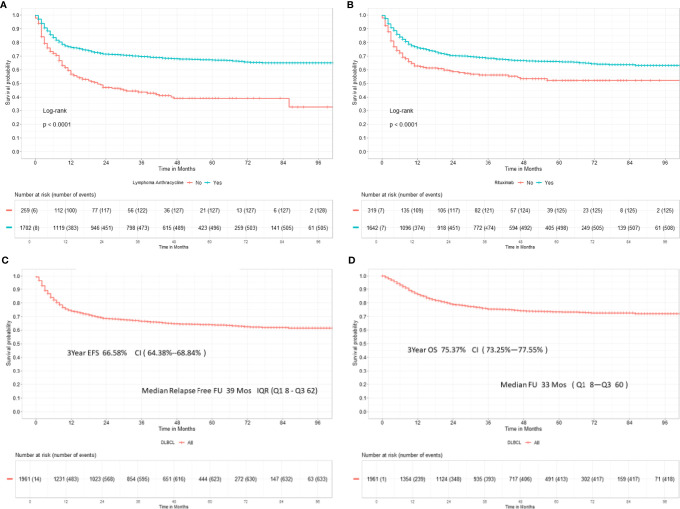
**(A)** Three-year event-free survival in 1,961 DLBCL patients receiving anthracyclines. **(B)** Three-year event-free survival in DLBCL patients receiving rituximab. **(C)** Three-year event-free survival in DLBCL. **(D)** Three-year overall survival in DLBCL.

The 3-year EFS for patients receiving anthracycline was 69.67% [95% CI, 67.40%–72.03%] and 43.81% [95% CI, 37.35%–51.39%] for those who did not receive anthracyclines (*p* < 0.0001), as shown in **Figure 3A**. Similarly, the EFS for patients receiving rituximab or not was 68.48% [95% CI, 66.14%–70.91%] and 56.22% [95% CI, 50.36%–62.76%], respectively, as shown in **Figure 3B**.

The EFS for the entire cohort at 3 years is 66.58% [95% CI, 64.38%–68.84%], as shown in **Figure 3C**. In total, 154/769 (20.03%) evaluable early-stage patients received reduced number of chemotherapy cycles (≤4). Consolidation radiotherapy was given to 47/154 (30%) early-stage patients. Also, 615/769 (79.97%) received standard chemotherapy with 6 cycles; 19.8% (122/615) of patients received post 6 cycles radiation. The 3-year EFS was 42.19% [CI, 34.57%–51.48%] versus 83.29% [80.19%–86.51%] for ≤4 versus 6 cycles of chemotherapy. Consolidation radiotherapy made a difference in the outcome of patients receiving ≤4 cycles of chemotherapy (3-year EFS 60.55% vs. 33.94%) but not for patients receiving 6 cycles of chemotherapy (75.99% vs. 85.30%). For advanced stage disease, the 3-year EFS was 70.30% for patients receiving chemotherapy alone and 61.60% for patients receiving consolidation radiotherapy post-6 or more cycles of chemotherapy (*p* = 0.074). The 3-year EFS for patients receiving daEPOCH-R was 55.57% [CI, 46.24%–66.30%].

The multivariate analysis suggests age <65 years, early stage, low-risk IPI, anthracycline and rituximab made an impact on the 3-year EFS (**Table 3**).

**Table 3:** Multivariate analysis.

Characteristic	coef	Hazard ratio	Lower CI	Upper CI	*p*-value
**Age ≥65 years**	−0.351	0.704	0.589	0.841	<0.001
**Stage**	0.238	1.268	1.167	1.378	<0.001
**IPI risk group**	−0.175	0.839	0.773	0.912	<0.001
**Chemotherapy group**	−0.102	0.903	0.565	1.443	0.67
**Anthracycline**	−0.614	0.541	0.295	0.992	0.047
**Rituximab**	−0.485	0.616	0.407	0.930	0.021

Due to lack of records of grades 1 and 2 toxicity in the EMR, the serious adverse events recorded were during hospitalization. Thirty-four patients were hospitalized for grade 3 or 4 febrile neutropenia. Early treatment mortality occurred in 27 patients (1.37%) due to infection, hemorrhage, or tumor lysis. In 4 patients, the cause of mortality was unrelated to treatment toxicity. A total of 124 patients (6.32%) discontinued treatment prior to mid-treatment response evaluation due to financial constraints and other social reasons.

On follow-up 1,325 patients remain in first remission at the last follow-up. In total, 408 patients who relapsed or progressed on treatment were offered salvage therapy; 226 patients (55.36%) underwent salvage therapy; and 22 (9.7%) opted for high-dose consolidation and autohematopoietic stem cell rescue (HSCT). The OS at 3 years was 75.37% [95% CI, 73.25%–77.55%], as shown in **Figure 3D**.

## Discussion

DLBCL is a curable disease. The survival rates in patients who remain disease free for 2 years after front-line therapy is similar to that of the general population (12, 13). The treatment landscape of DLBCL continues to evolve (14, 15). Real-world evidence (RWE) collected from different populations is crucial in demonstrating reproducibility of clinical trial results and targeting patient subgroups that are adequately represented in pivotal studies. RWE in DLBCL gathered in Europe and the USA have studied cancer registries or medical records of integrated healthcare networks (16–19). Indian literature on DLBCL has reports from single Institute data (2, 3, 20, 21), and collaborative registries on outcome are few (6). In reported studies, DLBCL is the commonest subtype of lymphoma accounting for 30% to 68% in the studies reported (2, 3, 20–22). The median age at diagnosis of DLBCLs in western literature is mid-60s and 30% are older than 75 years of age. Indian data from single institute suggested a lower median age (≤50 years) at diagnosis (**Table 4**). This effect is due to a younger population in the country and a bias of younger patients taking treatment at referral centers. More recent studies including the present data suggest the median age is 57 years, in centers which cater to the local population.

**Table 4:** Review of DLBCL presentation and outcomes from Indian studies.

Reference	2	3	6	21	Present study
**Time period**	2000–2013	2006–2015	2005–2009	2013–2015	2011–2017
**Number**	444	526	791	267	1,961
**Age (range)**	47 (15–60)	50 (6–83)	52 (16–92)	49 (20–81)	57 (18–89)
**Gender ratio**	1.8:1	2.09:1	2:1	2:1	1.6:1
**B’ symptoms**		42%	37.8%	45%	43.2%
**Stage**					
**I/II**	45%	64%	43%	52%	43%
**III/IV**	55%	36%	53%	48%	57%
**IPI-L/L-I**	50%	64.4%	L-44%	40%	70%
	34%		I-26.8%	45%	
**H-I/H**	16%	35.5%	H-24.5%	15%	30%
**Rituximab given**	27%	21%	42.7%	45%	83.73%
**Overall response**	82%		66%	84%	79
**CR**	75%		55%	70%	61.75%
**Survival**	Median, 46 months	Median, 22 months (CHOP, 21 months; R-CHOP, 33 months)		2-year EFS, 75% (CHOP, 61%; R-CHOP, 77%)	3-year EFS, 66.58%
	3-year OS, 76%			2-year OS, 70%	3-year OS, 75.37%

Over the last two decades, unique DLBCL subtypes by either cell of origin (COO) or molecular characterization have been identified (23). The capacity to perform gene expression profiling routinely is limited, and IHC algorithms are commonly used to determine the COO in clinical practice (8). GCB tumors express CD10 and or bcl-6 while the non-GCB subtypes express IRF4 and bcl-2. At diagnosis, determination of CCO is not yet the standard of care, and data available in the EMR records were available for less than half the patients. GCB subtype was determined by CD10 positivity. The long-term outcomes in this study did not suggest that outcomes differ for GCB and non-GCB. However, this result may be the result of suboptimal IHC classification of COO. Also, a large proportion of patients in this cohort were treated with rituximab and hence the difference in outcomes between GCB and non-GCB may not have been observed. In another recent study from India, of 71patients, the 2-year disease-free survival was 70% versus 53% in GCB versus non-GCB subtypes (*p* = 0.38) (24). Rituximab was used in 75% of the patients in the present study. More studies are needed to validate the role of COO in the outcomes of DLBCL.

IPI is used to predict the prognosis in aggressive NHLs treated with doxorubicin-containing regimens (9). This score has been validated in the rituximab era (R-IPI). IPI risk categories were validated in this cohort of patients as well. Staging bone marrow was positive in 15% to 20% of cases, and when discordant large B cells are present, it is associated with poor prognosis (25). Bone marrow biopsy is no longer mandatory in patients who have undergone PET-CT staging. Since CT scans was the preferred staging modality, 75% of patients underwent a staging bone marrow study with 19% positivity in this study. PET-CT is a valuable tool to accurately determine baseline staging in lymphoma; however, its use is limited to availability and financial constraints.

The key differences in presentation in India as compared with the west include a lower median age of ≤50 years at presentation, higher male-to-female ratio, higher proportion of patients with poor ECOG performance status at diagnosis, higher proportion of patients with high and intermediate IPI risk group, and more B’ symptoms (**Table 4**). The collaborative data in the OncoCollect registry has changed some of these perspectives with regard to median age being higher (57 years), improved gender ratio (1.6:1), and more patients in the low and low-intermediate IPI (70%).

The standard frontline treatment of DLBCL remains chemoimmunotherapy with R-CHOP with or without radiation according to disease, stage, and clinical risk factors. Early stage accounts for 30% of all DLBCL cases. SWOG S8736 trial which randomized patients with early-stage DLBCL-abbreviated chemotherapy (CHOPx3) plus consolidative radiation therapy over CHOPx8 showed similar PFS and OS (12 vs. 11.1 years, *p* = 0.73 and 13.0 vs. 13.7 years, *p* = 0.38) (26). With the addition of rituximab to CHOPx3-RT in SWOG S0014, the median PFS and OS have not been reached at a median follow-up of 12 years (27). PET-guided approach of abbreviated chemotherapy with or without radiation in early-stage DLBCL, nonbulky who achieved complete metabolic response shows comparable 5-year survival in radiation versus observation arm (28, 29). In the present study, the outcomes for abbreviated chemotherapy cycles for early-stage disease were inferior. This may suggest that reducing therapy must be limited to a highly selected group of patients who undergo adequate staging with PET-CT scans at diagnosis. More RWE is required before reducing chemotherapy cycles in early-stage disease. This is important since the option for salvage is available to approximately 50% of relapsed and refractory patients, and high-dose chemotherapy with HSCT is feasible in less than 10% patients.

The standard front-line treatment of advanced stage DLBCL remains R-CHOP for the last two decades (5). The majority of patients were treated with R-CHOP in this study, and the 3-year EFS at 72.85% is reasonable. Multiple attempts to improve the R-CHOP back bone including intensification of dose intensity (R-CHOP x 14 vs R-CHOP x 21), other CD 20 monoclonal antibodies (rituximab vs obinutuzumab) and infusional versus bolus (CALGB50303) have so far not translated into improved patient outcomes (30–32). A selected group of high-risk patients treated with daEPOCH-R in this study had a 3-year EFS of 55.37%.

Improving access to rituximab for patients as well as better supportive care to enable patients to tolerate full-dose therapy will improve results further. Biosimilar rituximab was first launched in India in 2007, and pharmacokinetics (33) and postmarketing clinical equivalence were established (34). The availability of multiple brands of biosimilar rituximab since 2015 has resulted in standard immunochemotherapy being available to more patients (2, 35). In this study, 16% of patients who were medically fit to take CHOP could not take rituximab for financial reasons. Significant number of these patients discontinued treatment, reflecting socioeconomic causes as the reason for discontinuation. Attempt should be made to give standard R-CHOP with financial support, which may motivate patients to complete treatment and improve outcomes. Since most patients spend “out of pocket” for treatment, the first chance for cure is the best chance and all attempts to complete treatment should be made.

Lack of access to specialized cancer centers, diagnostic delay, and suboptimal or inappropriate management compounded by socioeconomic factors were probably contributors to inferior outcomes in the past. The outcome of DLBCL in India can be improved with treatments in regional cancer center, structured data collection, centralized pathology review, training, uniform chemotherapy protocols, and financial and social support to DLBCL patients.

## Conclusion

DLBCL remains the commonest (44%) lymphoma subtype and is curable by standard anthracycline- and rituximab-based therapies. The availability of rituximab has increased the proportion of patients receiving standard chemoimmunotherapy. Further improvement in DLBCL outcomes largely depends on reducing dropouts during treatment and in the first 2 years of follow-up posttreatment completion. Regular audits can help innovate mechanisms in assisting treatment completion through patient access programs, improving follow-up in the first 2 years of treatment completion through social and financial support.

## Data Availability Statement

The raw data supporting the conclusions of this article will be made available by the authors, without undue reservation.

## Ethics Statement

The studies involving human participants were reviewed and approved by the Tata Medical Center-Institutional Review Board (TMC-IRB). Written informed consent for participation was not required for this study in accordance with the national legislation and the institutional requirements.

## Author Contributions

Concept and design: RNa, DB, SRaj, RNi. Literature search: RNa and DB. Clinical management: all clinicians mentioned in the contributors’ list except RNi. Data acquisition and data analysis: RNi, Institute IT teams, and clinical coordinators. manuscript preparation: RNa, DB, SRaj, and RNi. Manuscript editing: SRaj, AK, RB, SS, HM, SRam, AS, and RNi. Manuscript review: RNi. IA guarantors: RNa and RNi. All authors contributed to the article and approved the submitted version.

## Conflict of Interest

Author SRam was a consultant for Kumaran Hospital (P) Ltd. (MCCF).

The remaining authors declare that the research was conducted in the absence of any commercial or financial relationships that could be construed as a potential conflict of interest.

## Publisher’s Note

All claims expressed in this article are solely those of the authors and do not necessarily represent those of their affiliated organizations, or those of the publisher, the editors and the reviewers. Any product that may be evaluated in this article, or claim that may be made by its manufacturer, is not guaranteed or endorsed by the publisher.
